# Hepatic stellate cells increase in *Toxoplasma gondii* infection in mice

**DOI:** 10.1186/1756-3305-6-135

**Published:** 2013-05-04

**Authors:** Hasan Tarık Atmaca, Aycan Nuriye Gazyagcı, Sıla Canpolat, Oguz Kul

**Affiliations:** 1 Permanent address: Department of Pathology, Faculty of Veterinary Medicine, University of Kırıkkale, Kırıkkale 71451, Turkey; 2 Present address: Department of Pathology, Faculty of Veterinary Medicine, Kyrgyzstan-Turkey Manas University, Bishkek 720044, Kyrgyzstan; 3 Department of Parasitology, Faculty of Veterinary Medicine, University of Kırıkkale, Kırıkkale 71451, Turkey

**Keywords:** *Toxoplasma gondii*, Liver, Hepatic stellate cell, Hepatitis, Acute

## Abstract

**Background:**

*Toxoplasma gondii* is a ubiquitous protozoan parasite that can infect humans and animals. The severity of toxoplasmosis varies according to the immune status of the individual, parasite strain, and host species. In mammalian species, it has been observed that severe lesions of acute toxoplasmosis form in visceral organs such as the liver, lung, and spleen. Some epidemiological studies have reported an association of *T. gondii* infection with liver cirrhosis.

**Methods:**

Acute infection was induced in fifteen 30-day-old normal Swiss albino mice. The mice were infected by intraperitoneal inoculation of 5000 *T. gondii* RH strain tachyzoites. The mice were sacrificed in groups of 5 at 2, 4, and 6 days after inoculation. Another group of 5 mice were used as the controls. Anti-glial fibrillary acidic protein (GFAP) and anti-*T*. *gondii* antibodies were used to compare GFAP-immunoreactive cells and anti-*T. gondii*–immunopositive areas in the liver between the *T. gondii*-infected groups and the healthy controls, respectively.

**Results:**

There was a significant correlation between the numbers of GFAP-positive hepatic stellate cells (HSCs) when they were compared with *T. gondii* antigen immunostaining (p < 0.05). The amount of *T. gondii* immunostaining increased significantly with the increase in the number of HSCs.

**Conclusions:**

There is a significant relationship between the number of HSCs and *T. gondii* antigens, which may represent an active role of HSCs in liver pathology and the pathobiology of *T. gondii*-related hepatitis.

## Background

Toxoplasmosis is a parasitic disease caused by the protozoan *Toxoplasma gondii.* It can infect humans and animals [1,2]. Typically, infection occurs after ingestion of raw or undercooked meat containing tissue cysts or through accidental environmental contamination by oocysts. Only a small percentage of humans and animals display clinical symptoms following infection. The severity of toxoplasmosis varies according to the immune status of the individual, parasite strain, and host species [2-5]. Severe lesions due to acute toxoplasmosis have been observed in visceral organs such as the liver, lung, and spleen of mammalian species [6]. It is known that the parasite locates to the host liver, lymph nodes, eyes, heart, and central nervous system, causing the lesions. In the liver, it causes pathological changes that progress to hepatomegaly, granuloma, hepatitis, and necrosis. Some epidemiological studies have reported an association of *T. gondii* infection with liver cirrhosis [7]. An important functional role of stellate cells in liver repair as indicated by the close proximity of this cell type to collagen fibers in injured livers [8]. Hepatic stellate cells are known to play an important role in the development of fibrosis and its advancement to cirrhosis. Activated hepatic stellate cells differentiate from vitamin-A-storing cells to contractile myofibroblastic cells responsible for extracellular matrix (ECM) production in the damaged liver. There is rising evidence mainly from the experimental studies that HSCs acquire signaling pathways required for stem/progenitor cell functions [9]. Hepatic stellate cells can also interact with the sinusoidal space, where other antigen presenting cells (APC) such as dentritic cells and liver macrophages or kupffer cells are existent. Therefore, HSC might also affect the Ag-presenting function of these APC [10].

The liver consists of parenchymal and non-parenchymal cells. The non-parenchymal cells include endothelial cells, Kupffer cells, liver-associated natural killer cells, hepatic stellate cells (HSCs), and cholangiocytes [11].

HSCs are defined as mesenchymal cells. These cells contain cytoskeletal proteins such as vimentin, α-smooth muscle actin (α-SMA), desmin, and glial fibrillary acidic protein (GFAP) [12,13]. In this study, we used GFAP to identify HSCs.

To our knowledge, the role of HSCs in acute hepatic lesions caused by *T. gondii* infection has not been investigated before. In this study, we demonstrated the role of HSCs in *Toxoplasma* hepatitis using immunohistochemical tests.

## Methods

### Study design

Twenty 30-day-old normal Swiss albino mice, weighing approximately 25–30 g, were used to study acute *T. gondii* infections. They were supplied by, The Turkish Public Health Institution. The virulent *T. gondii* RH strain, which is maintained in the Parasitology Laboratory of the Turkish Public Health Institution by continuous passage every 3–4 days in mice, was used to induce acute infection. Fifteen mice were infected by intraperitoneal inoculation of 5000 *T. gondii* RH tachyzoites. In groups of 5, the mice were sacrificed at 2, 4, and 6 days after inoculation (DAI). Another healthy 5 mice sacrificed at the beginning of study, were used as the control in the immunohistochemical tests. The Animal Care Committee of the University of Kırıkkale approved this study. (permission date:11.02.2011, no. 11).

### Necropsy and histopathology

Mice were sacrificed by cervical dislocation after they had been anesthetized with pentobarbital. The livers were removed and fixed in 10% formaldehyde in 0.1 M phosphate buffer. The fixed tissue was dehydrated and embedded in paraffin. The livers were sectioned to 5-μm thickness and stained with hematoxylin and eosin (H&E).

### Immunohistochemistry

HSCs were stained for immunohistochemistry (IHC) using 1:50 rabbit polyclonal anti-GFAP antibody (Thermo Fisher Scientific Inc., USA). *Toxoplasma* antigens were demonstrated as described previously [14] using 1:100 rabbit anti-*T. gondii* antibody. All immunohistochemical tests were performed according to a commercial streptavidin–biotin kit (Thermo Fisher Scientific Inc.) protocol that used diaminobenzidine (DAB) as a chromogen and Mayer’s hematoxylin as a counterstain. Mouse brain experimentally infected with the *T. gondii* Me49 strain served as a positive internal control for the GFAP and *T. gondii* antigens. The negative control underwent the same process, but in the absence of the primary antibody during the staining procedure. Sections on poly-l-lysine–coated slides were deparaffinized. Endogenous peroxidase blocking was carried out using 3% hydrogen peroxide in methyl alcohol for 10 min. Heat retrieval was carried out using Tris buffer solution (TBS, pH 6.0) in a pressure cooker. The protein blocking stage spanned 7 min. Treatment with the primary antibody (anti-GFAP or anti-*T. gondii*) on the positive control slides was carried out for 1 h, followed by secondary antibody for 30 min, and finally with streptavidin for 30 min. Slides were rinsed with TBS (2 × 5 min) after each step. Sections were treated with DAB for 10 min and counterstained with Mayer’s hematoxylin. Microphotographs were captured using an Olympus BX51 microscope with a DP25 camera attachment (Japan).

### Histomorphometric analysis and statistics

Leica QWin Plus image processing and analysis software (Leica, Germany) was used to evaluate the intracellular and interstitial immunostaining. Briefly, at least 5 randomly selected and consecutive × 20 objective microscope fields were photographed. After calculation of the proportional (% pixels) staining area to whole fields, the mean % pixels staining area for each slide was calculated. To evaluate non-parametric data, the Mann–Whitney *U*-test was used to compare GFAP-immunoreactive cells and anti-*T. gondii*–immunopositive areas in the liver between the *T. gondii*-infected groups and the healthy controls. The relationship between the HSCs and *T. gondii* antigens were assessed using the Kruskal–Wallis test. A p-value of <0.05 was considered statistically significant.

## Results

### Histopathology

**Figure 1:**
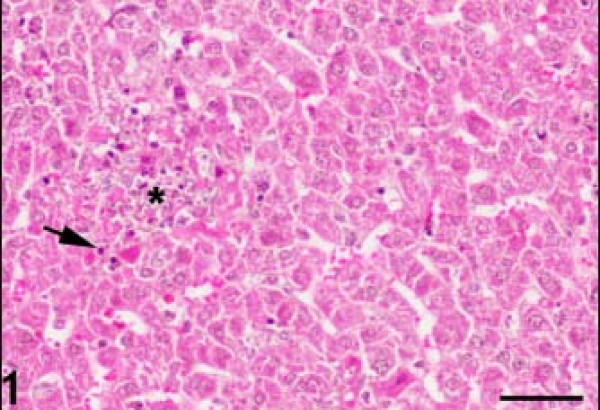
**Foci of hepatocellular necrosis (asterisk) and mononuclear cell infiltration (arrow).** Hematoxylin and eosin staining. Bar: 50 micrometer.

**Figure 2:**
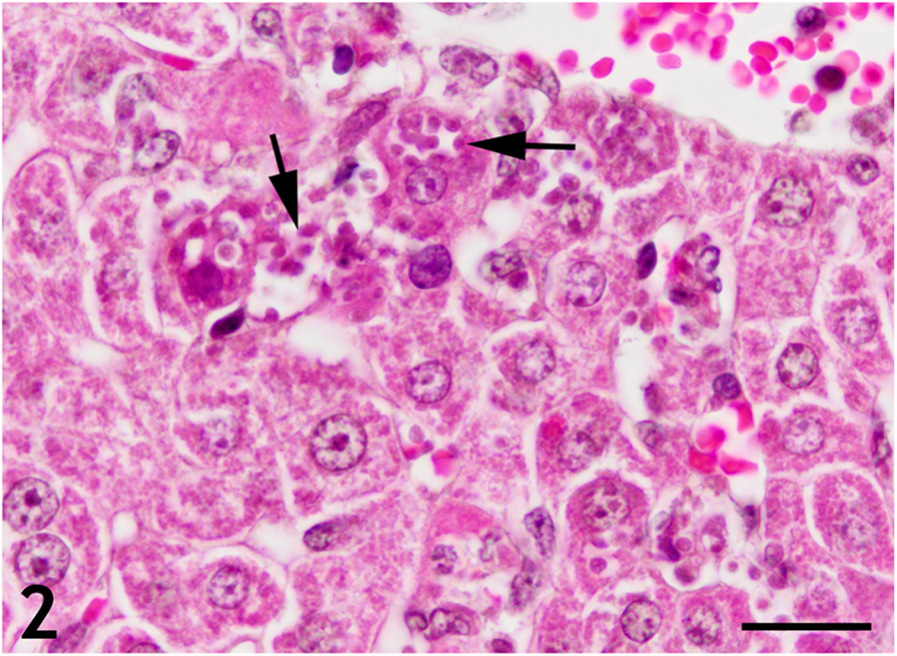
**Hepatocytes filled with large groups of parasites (arrows). Hematoxylin and eosin staining.** Bar: 20 micrometer.

**Figure 3:**
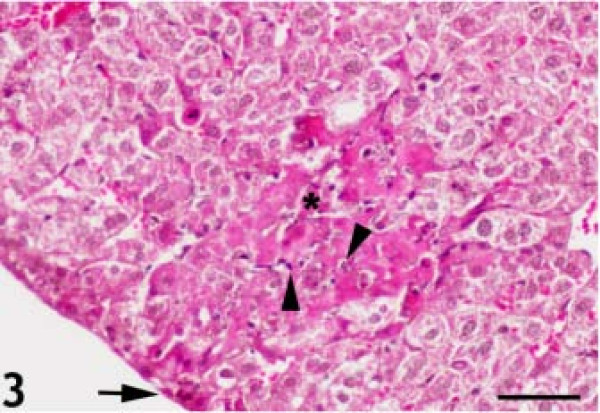
**Karyorrhexis and karyolysis in the nuclei of necrotic hepatocytes (arrowheads).** There were several small inflammatory lesions on the surface of the liver (arrow); the number of lesions increased and progressed into deeper areas (asterisk). Hematoxylin and eosin staining. Bar: 50 micrometer.

The control H&E-stained liver sections exhibited normal hepatic architecture. In *T.gondii* infected mice, there were multiple foci of hepatocellular necrosis and mononuclear cell infiltrations (Figure 1). Mild lymphocytic and plasma cell infiltrations were found in the periportal areas and around the central veins. A few tachyzoite clusters were present in the cytoplasm of the hepatocytes. Some hepatocytes were filled with a large group of parasites (Figure 2). Karyorrhexis and karyolysis were observed in the nuclei of necrotic hepatocytes (Figure 3).

Several small inflammatory lesions were present on the surface of the liver, increasing in number and progressing into deeper areas over time after inoculation (Figure 3). While it was difficult to identify individual parasites in tissue sections at 2 DAI, the parasites were easily identified after immunohistochemical staining. At 4 and 6 DAI, parasite clusters became apparent in both hepatocytes (H&E) and stellate cells (GFAP, IHC). Small numbers of parasites were associated with the connective tissue on the surface of the liver during the early stages of infection at 2 DAI. There was a massive increase in the numbers of parasites associated with the connective tissue at 4 and 6 DAI as compared to that at 2 DAI. The parasites were also present along the connective tissue strands within the liver. The initial inflammatory lesions contained few parasites, but these increased rapidly over time, becoming numerous at 4 DAI. In addition, a number of hepatocytes and sinusoidal cells were infected, and a number of these contained large groups of parasites.

### *T. gondii IHC*

**Figure 4:**
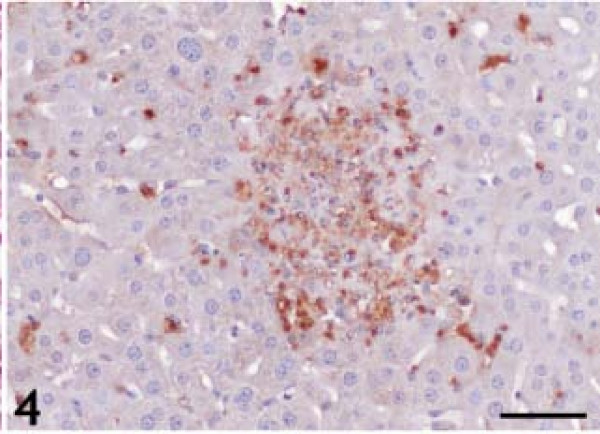
**Anti-*Toxoplasma gondii *antigen immunopositivity throughout the liver, in necrotic foci, and individual hepatocytes.** Diaminobenzidine chromogen and hematoxylin counterstain. Bar: 50 micrometer.

**Figure 5:**
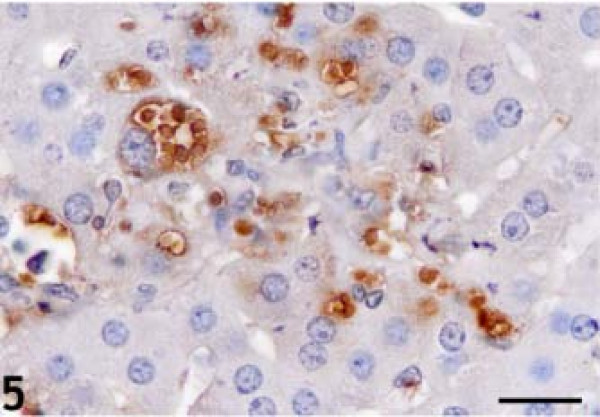
**Anti-*Toxoplasma gondii *antigen immunopositivity in individual hepatocytes.** Diaminobenzidine chromogen and hematoxylin counterstain. Bar: 20 micrometer.

The statistical evaluation of *T. gondii* antigen expression in the liver of *T. gondii*–infected and control animals is listed in Table 1. *T. gondii* antigens were detected in all infection groups. At 2 DAI, positive immunoreactions were observed at the surface and underside of Glisson’s capsule. At 4 and 6 DAI, necrotic foci, hepatocytes, and HSCs containing tachyzoites were *T. gondii* antigen–immunopositive (Figures 4 and 5). *T. gondii* antigen immunoreactivity was not detected in specific locations. Immunopositive reactions were observed throughout the lobules.

**Table 1:** **GFAP and *T. gondii *immunohistochemical test results and statistical data**

**Antibody**	**Control (n = 5)**		**2 DAI (n = 5)**		**4 DAI (n = 5)**		**6 DAI (n = 5)**		Statistical significance p*
	**Mean**	**Std. deviation**	**Mean**	**Std. deviation**	**Mean**	**Std. deviation**	**Mean**	**Std. deviation**	p**
GFAP	0.902	0.080	2.515	0.383	3.090	0.171	3.609	0.316	<0.05
*T. gondii*	0	0	3.538	0.440	4.136	0.218	4.632	0.337	<0.05

*DAI* days after infection, *GFAP* Glial fibrillary acidic protein, *T. gondii Toxoplasma gondii.*

*Mann–Whitney test.

**Kruskal-Wallis test.

### HSCs detected by GFAP IHC

The statistical evaluation of GFAP antigen expression in the HSCs of *T. gondii*-infected and control animals are listed in Table 1. The number of HSCs was highest (3.609) in the 6 DAI group as compared to 3.090, 2.515, and 0.902 in the 4 DAI, 2 DAI, and control groups, respectively (p < 0.05). There was a significant correlation between the numbers of GFAP-positive HSCs when they were compared with *T. gondii* antigen immunostaining (p < 0.05). The amount of *T. gondii* immunostaining increased significantly along with the increase in the number of HSCs (Table 1).

**Figure 6:**
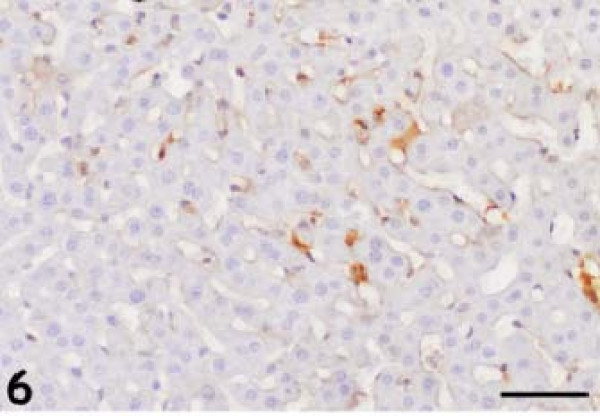
**The anti-glial fibrillary acidic protein antibody produced thin, irregular immunopositive bands, which lined the hepatic sinusoids of the control animals.** Diaminobenzidine chromogen and hematoxylin counterstain. Bar: 50 micrometer.

**Figure 7:**
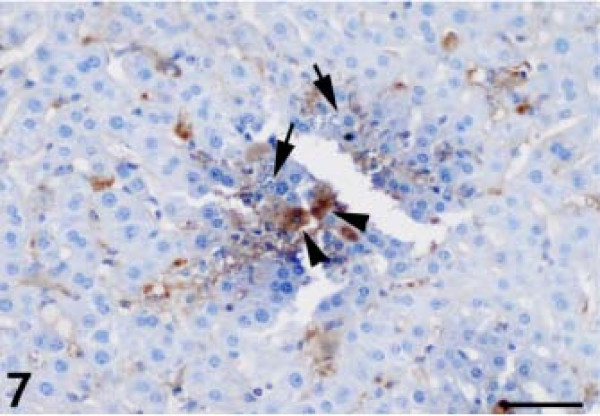
**Anti- glial fibrillary acidic protein (GFAP)–positive hepatic stellate cells containing parasites (arrowheads); parasite-containing hepatocytes (arrows) did not react with the anti-GFAP antibody.** Diaminobenzidine chromogen and hematoxylin counterstain. Bar: 50 micrometer.

**Figure 8:**
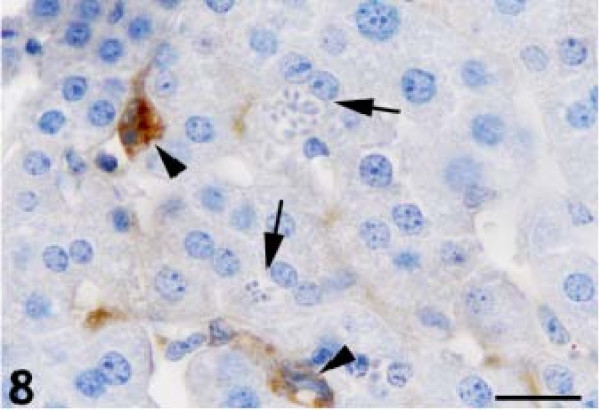
**Anti-glial fibrillary acidic protein–positive hepatic stellate cells and sinusoids containing parasites (arrowheads), parasite-containing hepatocytes (arrows).** Diaminobenzidine chromogen and hematoxylin counterstain. Bar: 20 micrometer.

The anti-GFAP antibody produced thin, irregular immunopositive bands that lined the hepatic sinusoids of the control animals (Figure 6). GFAP-positive HSCs were present within the perisinusoidal space of Disse in the liver, which is lined by parenchymal cells and fenestrated sinusoidal endothelial cells (SECs). We observed a significantly higher number of GFAP-positive HSCs at 2, 4, and 6 DAI, with activated stellate cells in the periportal, intermediate, and periacinar areas (Figure 7). The GFAP-positive HSCs also contained numerous proliferating parasites (Figure 8).

## Discussion

To our knowledge, this is the first experimental study of acute *T. gondii* infection analyzing HSC activation and its correlation with *T. gondii*-associated lesions in the liver. We carried out immunohistochemical analysis of the number of activated HSCs as detected by GFAP staining. The number of activated HSCs was significantly higher in the *T. gondii* infection groups than that in the healthy group.

Acute liver injury was followed by an almost exclusive increase in the number of HSCs, while HSCs as well as liver myofibroblasts were involved in scar formation in chronically injured livers [15].

Intravital microscopy imaging studies have determined that apicomplexan parasites such as *T. gondii* invade the liver, gradually move toward the surface of sinusoidal epithelial cells to the Kupffer cells, finally entering and developing in the cytoplasm of hepatocytes [16].

GFAP expression decreases upon HSC activation, whereas desmin and α-SMA levels increase due to HSC transformation into myofibroblast-like cells [17]. In this study, increased GFAP immunopositive HSCs were related to the *Toxoplasma gondii* infection in the mice. Additionally, the increased GFAP immunopositivity in the lesions and perisinusoidal region suggests the accumulation of HSCs at locations of active initial fibrogenesis.

The nature of stellate cells, traditionally considered cells of mesenchymal origin due to their morphologic features and their expression of molecules such as vimentin, desmin, and α-SMA, has been questioned recently, as numerous studies have clearly demonstrated the expression of neural and neuroendocrine markers such as GFAP, nestin, neurotrophin receptor, and synaptophysin [18].

In the normal liver, HSCs comprise approximately 1.4% of the total liver volume and are present at a ratio of approximately 3.6–6 cells per 100 hepatocytes (1:20). HSCs are typically located in the perisinusoidal space of Disse, a recess between SECs and hepatocytes [18]. We demonstrated activated HSCs using a specific polyclonal antibody to detect GFAP. GFAP is an early marker of stellate cell activation in the liver [19] and has been suggested as a more reliable marker for perisinusoidal stellate cells than desmin [20]. Buniatian *et al.* showed both GFAP and vimentin were strongly expressed in the perinuclear region and the cell processes of cultured stellate cells. They also demonstrated staining for GFAP highly overlapped with that for vimentin or desmin in cultured stellate cells and with that for vimentin in the liver sections. Desmin-positive cells were always also GFAP-positive [20].

However, of the GFAP-positive cells only an estimated 50% were found desmin-positive. In addition, GFAP has been established as one of several markers for identifying HSCs [21].

Ueberham *et al.* were the first to demonstrate the proliferation of midzonal and pericentral HSC populations [22]. They demonstrated the proliferative state of distinct sinusoidal cell populations by double-labeling experiments, merging bromodeoxyuridine staining with GFAP in the liver sections of choline-deficient, ethionine-treated mice. The increase of HSCs in *Toxoplasma* hepatitis can be described easily. The number of HSCs detected increased at 2 DAI, and continued to increase at 4 and 6 DAI. These data are analogous to the *T. gondii* antigen. This means that as the amount of *T. gondii* antigen increased, the number of HSCs and histopathological lesions in the liver increased in tandem.

An increasingly complex interplay between various cell types in the liver and stellate cells has become apparent. Hepatocytes, Kupffer cells, platelets, epithelial cells, SECs, and neutrophils are known to interact with stellate cells. Each of these cell types will release a subset of mediators that have diverse effects on HSCs [18]. We are curious as to whether HSCs also release mediators. Future studies may help to determine or describe the interaction of cytokine or mediator-releasing HSCs in liver disease, including *T. gondii* infection. Moreover, double immunohistochemical staining, that can be defined as the detection of two or more targets on one slide, thus increasing the information obtained from each tissue section, can be performed on HSCs.

It is widely accepted that HSCs contribute to liver fibrosis. HSCs reside within the perisinusoidal space of Disse in the liver lined by parenchymal cells and fenestrated SECs. HSCs can develop into myofibroblast-like cells that synthesize extracellular matrix proteins and contribute to liver fibrosis [8,23-25].

Another experimental study has yielded similar information, determining that HSCs are directly involved in the restoration of liver mass even *in vivo*[26]. Rastogi *et al.*[9] reported a possible dynamic role of HSCs in liver regeneration and the pathobiology of acute-on-chronic liver failure. Our study also highlights the probability that HSCs play an important role in liver regeneration. This is because the proliferation of parasites in HSCs and the number of HSCs increase in parallel. This may indicate the beginning of liver regeneration with HSCs. We do not know how HSCs can play a role in treated liver injury from this study. After successful treatment of *T. gondii* infection in liver, we would like to see the regeneration and the status of hepatic stellate cells.

Future studies in liver diseases of different etiologies and stages using immunohistochemical markers and molecular studies of HSCs are needed to confirm these initial observations, and might provide further insight into liver injury and repair mechanisms and may help to improve therapeutic interventions in liver diseases associated with *T. gondii*.

## Conclusions

Thus, the number of activated HSCs in hepatitis due to *T. gondii* was found to be higher than healthy liver. There is a significant relationship between the number of HSCs and *T. gondii* antigens, which may represent an active role of HSCs in liver pathology and the pathobiology of *T. gondii*-related hepatitis. In addition, the findings from this study would be useful for investigations of experimentally induced liver lesions in mice.

## Competing interests

The authors declare that they have no competing interests.

## Authors’ contributions

HTA conceived the study and wrote the manuscript. ANG and SC performed the experiment. OK assisted with the statistics. All authors read and approved the final manuscript.
